# The therapeutic role of Jingchuan tablet on ischaemic cerebral stroke via the HIF-1α/EPO/VEGFA signalling pathway

**DOI:** 10.1080/13880209.2022.2134430

**Published:** 2022-10-21

**Authors:** Yan Zhang, Qinghuan Liu, Ting Zhang, Hong Wang, Yu Fu, Wentong Wang, Dongdong Li

**Affiliations:** Tianjin Institute of Medical and Pharmaceutical Sciences, Tianjin, China

**Keywords:** Cerebral ischaemia, neuronal apoptosis, network pharmacology, molecular mechanism, traditional Chinese medicine

## Abstract

**Context:**

Jingchuan tablet (JCT) is a Chinese medicine prescription for treating ischaemic cerebral stroke (ICS). However, its relevant mechanisms remain unclear.

**Objective:**

To unravel the intrinsic mechanisms of JCT anti-ICS.

**Materials and methods:**

‘Hongjingtian’, ‘chuanxiong’, ‘yanhusuo’, ‘bingpian’, ‘cerebral infarction’, ‘cerebral ischemia’ or ‘stroke’ were used as keywords, and then components, targets and underlying mechanisms of JCT anti-ICS were analysed in TCMSP, TTD, DrugBank, STRING and Metascape databases up to June 2020. Male Sprague-Dawley rats under permanent middle cerebral artery occlusion (pMCAO) model, randomly assigned as: model, sham, nimodipine (0.012 g/kg/d) and JCT (0.78, 1.56 and 3.12 g/kg/d) groups, received oral gavage administration for a week. Therapeutic effects were evaluated by detecting the proportion of cerebral infarction, neuronal apoptosis and neurological deficits. Bioactive components were detected by HPLC–MS. Molecular biology and computational docking were used to verify the underlying mechanisms.

**Results:**

Eighty-one components, 166 targets and HIF-1α/EPO/VEGFA pathway contributed to the anti-ICS effect of JCT. JCT treatment effectively reduced the proportion of cerebral infarction (33.13%), apoptosis rate (14.80%) and neurobehavioural score (2.00). JCT increased the protein levels of HIF-1α (0.84), EPO (0.64) and VEGFA (0.69), respectively (*p* < 0.05). Gallic acid, salidroside, chlorogenic acid, ethyl gallate, ferulic acid and tetrahydropalmatine detected by HPLC–MS showed good interaction and binding with HIF-1α/EPO/VEGFA.

**Conclusions:**

Our study demonstrated the mechanisms of JCT anti-ICS associated with the activation of the HIF-1α/EPO/VEGFA pathway, which provided a pharmacological basis for expanding the clinical application and some scientific ideas for further research into the material basis JCT anti-ICS.

## Introduction

Stroke is the second most fatal disease in the world and has a high disability rate (Johnson et al. 2019). In stroke cases, ischaemic cerebral stroke (ICS) accounts for 87% (Zhu et al. 2022). Cerebral ischaemia causes various damage to the cerebral microcirculation, such as oxidative stress, insufficient capillary perfusion capillary hypoperfusion and destruction of brain–blood barrier (Lerouet et al. 2002; Cao et al. 2022). There is a critical need for promising therapeutic strategies for ischaemic cerebral injury.

Traditional Chinese medicine (TCM) is a summary of the extremely rich experience accumulated by the Chinese people in the long-term struggle against diseases. After many years of clinical application, TCM has become an important means of prevention and treatment of ICS (Wang et al. 2020; Zhu et al. 2022). Jingchuan tablet (JCT) is a safe and effective TCM for treatment of ICS developed by Tianjin Institute of Medical and Pharmaceutical Sciences, authorized invention patent (Tao et al. 2017). Under the guidance of TCM theory, the prescription treats ischaemic stroke based on the principle of ‘activating blood circulation, removing blood stasis and dredging collaterals’ (Liu et al. 2017; Wang et al. 2020) and it consists of *Rhodiola crenulata* (Hook. f. et Thoms.) H. Ohba (Crassulaceae) (HJT), *Corydalis yanhusuo* (Y. H. Chou et Chun C. Hsu) W. T. Wang ex Z. Y. Su et C. Y. Wu (Papaveraceae) (YHS), *Ligusticum chuanxiong* Hort. (Umbelliferae) (CX) and Borneolum syntheticum (BP) (Wang et al. 2017; Liu et al. 2018; Zhang et al. 2019). As a monarch herb, HJT is used for stroke caused by blood stasis and collateral stasis (Ran et al. 2019; Zhuang et al. 2019; Li et al. 2021). YHS and CX are the minister herbs, together with the monarch herb to enhance the function of promoting blood circulation and relieve the symptoms of stroke caused by blood stasis (Chinese Pharmacopoeia Commission (CPC) 2015; Tian et al. 2020; Zeng et al. 2021). BP is the adjuvant and guide herb. It has the function of opening up the orifices, clearing away heat and relieving pain, which is used as an assistant in stroke treatment to produce a synergic therapeutic effect (Chinese Pharmacopoeia Commission (CPC) 2015; Yu et al. 2021).

In our previous study, the acute cerebral ischaemia and normal pressure hypoxia tolerance experiments in rats confirmed that the anti-ICS effects of JCT were better than that of the single component herb (Tao et al. 2017; Liu et al. 2018), and found that the protective effects of JCT on ischaemic brain injury were related to HIF-1α, but it was uncertain whether HIF-1α was a direct therapeutic target (Zhang et al. 2019). Furthermore, we established a new and reliable quality control method for JCT by UPLC-ESI-MS/MS (Zhang et al. 2018). However, the effective ingredients, main therapeutic targets and molecular mechanisms of JCT against ICS are still unclear. Hence, it is necessary to conduct further research by means of network pharmacology and modern pharmacology.

Network pharmacology based on systems biology and multidirectional pharmacology has the systemic and holistic features, which is consistent with the synergistic effect of the multi-components of TCM (Jing et al. 2019; Zhou et al. 2020). It provides a new method for the comprehensive study of the active components and multi-target mechanisms of TCM, especially for the study of Chinese medicine prescription (He et al. 2021; Zhang et al. 2022). In this study, for clarifying the complex interaction of component-target-pathway in JCT, we performed network pharmacology to systematically investigate the effective components, potential targets and underlying mechanisms of JCT anti-ICS. After that, *in vivo* experiments were carried out to elucidate the complicated therapeutic effects of JCT on ICS. Then, mass spectrometry analysis was applied to help verify bioactive components in the target organ. Computational docking analysis and molecular biology were used for final mechanism verification. In general, this study adopted a comprehensive research model that combined bioinformatics, animal experiments, mass spectrometry analysis and molecular biology, which provided some ideas for explaining the mechanism of TCM prescriptions.

## Materials and methods

### Identifying component targets in JCT

JCT was provided by Tianjin Institute of Medical and Pharmaceutical Sciences (Tianjin, China). Specification: 0.5 g/piece. Batch number: 171225. The contents of gallic acid, salidroside, chlorogenic acid, tetrahydropalmatine, ferulic acid and levistolide A in JCT were 1.36, 3.86, 0.15, 0.07, 0.21 and 0.05 mg/g, respectively (Zhang et al. 2018). JCT was made of HJT, YHS, CX and BP. The origin of HJT was Tibet, China. The origin of YHS was Zhejiang, China. The origin of CX was Sichuan, China. The origin of BP was in Hebei, China. All the medicinal materials were purchased from Tianjin Traditional Chinese Medicine Decoction Pieces Factory (Tianjin, China). All samples were kept in the sample room of Tianjin Institute of Medical and Pharmaceutical Sciences (Tianjin, China).

The active components of the four herbs were screened from the Traditional Chinese Medicine Systems Pharmacology (TCMSP, http://tcmspw.com/tcmsp.php/) database, Integrative Pharmacology-based Research Platform of Traditional Chinese Medicine v2.0 (TCMIP, http://www.tcmip.cn/TCMIP/index.php/Home/) database and related literatures (Wang et al. 2007; Sun et al. 2012; Chen and Feng 2013; Mudge et al. 2012; Chung et al. 2017; Ma et al. 2017; Nan et al. 2018). Oral bioavailability (OB) and drug likeness (DL) were used to search the candidate active components from the TCMSP and TCMIP databases. For components in the TCMIP database, their OB and DL were evaluated in the ADMETlab (https://admet.scbdd.com/home/index/#) platform. The thresholds were set as OB ≥30% and DL ≥0.18 (Cui et al. 2020; Zhang et al. 2022). The structures of components were collected from the PubChem (https://pubchem.ncbi.nlm.nih.gov/) database. Then, the targets of components were found from the TCMSP database, TCMIP database, DrugBank (https://www.drugbank.ca/) database, SwissTargetPrediction (http://www.swisstargetprediction.ch/) database and related literatures. Afterward, the component targets in JCT were standardized in the UniProt (http://www.uniprot.org/) database.

### Identifying targets related to ICS

The targets related to ICS were searched from the TCMSP, TTD (http://db.idrblab.net/ttd/) and DrugBank databases by following keywords: ‘cerebral infarction’, ‘cerebral ischemia’ or ‘stroke’.

### Network construction

By comparing the component targets of JCT with the disease targets, we identified the possible active ingredients in JCT and their therapeutic targets for ICS. Then component-disease crossover genes were filtered on the VENNY 2.1 website (https://bioinfogp.cnb.csic.es/tools/venny/index.html/). Finally, Cytoscape 3.7.2 software was employed to construct the ‘drug-component-target-disease’ network.

### Network analysis

The protein–protein interactions (PPIs) were obtained and PPIs network analysis model was constructed by integrating the STRING 11.0 (http://string-db.org/) database and GenCLiP 3 (http://ci.smu.edu.cn/genclip3/analysis.php/) database. The gene ontology (GO) enrichment analysis was carried out in the Metascape (http://metascape.org/gp/index.html#/main/step1/) database using Multiple Gene List analysis, choosing *H. sapiens* species and setting *p* value cut-off to 0.01. Then, the potential targets of JCT were analysed from four aspects: cellular component (CC), molecular function (MF), biological process (BiP) and Kyoto Encyclopedia of Genes and Genomes (KEGG) pathway. Finally, the results of network analysis were visualized by using bioinformatics tools and the software of Cytoscape 3.7.2.

### Experimental animals and groups

Sixty male Sprague-Dawley rats (SPF, weight 160–180 g, approval no. SCXK 2016-0006) were obtained from Beijing Vital River Laboratory Animal Technology Co., Ltd. (Beijing, China). According to the result of pre-experiment and the number of samples needed to complete the follow-up test, 10 samples were allocated to each group. Rats were randomly divided into six groups according to the principle of completely random digital table, as follows: model (distilled water), sham (distilled water), positive drug control (nimodipine at a dosage of 0.012 g/kg; Tianjin Central Pharmaceutical Co., Ltd., Tianjin, China; specification: 0.03 g/piece; batch number: 171102), JCT-L (JCT at 0.78 g/kg), JCT-M (JCT at 1.56 g/kg) and JCT-H (JCT at 3.12 g/kg) groups. All rats had free access to a standard diet and drinking water, and they were fed in a room at 24.0 ± 0.5 °C with a 12 h cyclic lighting schedule. From the day of successful modelling, distilled water, positive drug and JCT were administered orally by gavage once daily for a week. After a week of treatment, the rats were anaesthetized with pentobarbital sodium (100 mg/kg, intraperitoneal injection) for euthanasia, and then brain tissues and blood samples were collected. The experiment was performed in compliance with the Animal Ethics Committee of Tianjin Institute of Medical and Pharmaceutical Sciences (IMPS-EAEP-Z-17JCYBJC28700-01) (Tianjin, China).

### The rat model of permanent middle cerebral artery occlusion (pMCAO)

The pMCAO model was established by modified Longa method (Ma et al. 2018; Wang et al. 2018). Rats were fasted for 12 h before operation. Anaesthesia was then performed with 10% chloral hydrate (3.5 mL/kg; Qingdao Yulong seaweed Co., Ltd., Qingdao, China). The left common carotid artery (CCA), external carotid artery (ECA) and internal carotid artery (ICA) were carefully exposed. The CCA and ECA were ligated with 5-0 thread at the bifurcation of the CCA/ECA. A small incision was made about 0.5 cm in the CCA away from the CCA/ECA bifurcation, and a wire bolt device (2432-A4 pipe, Beijing Cinontech Co., Ltd., Beijing, China) was inserted the incision until slight resistance was felt. Subsequently, the ICA was ligated and the operation was completed. After the rats woke up for 2 h, the mental state of the rats was observed and the neurobehavioural score was performed. When Horner’s syndrome occurred (Lin et al. 2017) and the neurobehavioural score was 1–3 according to the Longa scoring method (Longa et al. 1989), the model preparation was considered successful. The rats in the sham group were performed the same procedure without a bolt occlusion.

### Neurological behaviour assessment

After 48 h of modelling, two investigators blinded to group assignments observed the rats for symptoms and assessed neurological function (Ma et al. 2018). The neurological deficits of rats in each group were measured and scored by the Longa scoring method (Longa et al. 1989). The criteria were as follows: (1) normal activity without neurological symptoms (0 point); (2) unable to fully extend the contralateral forelimb (one point); (3) unable to extend the opposite forelimb (two points); (4) slightly circling to the opposite side (three points); (5) tilting to the opposite side (four points); (6) unable to walk spontaneously and lose consciousness (five points). Higher scores indicated more severe neurological deficits.

### The ratio of cerebral infarction

The brain tissues sections were stained with 1% TTC (2,3,5-triphenyl-2H-tetrazolium chloride; product batch number: 0412A18; Tianjin Ruijinte Chemical Co., Ltd., Tianjin, China) to observe the infarct state of rat brain tissue. Accurately weighed the quality of the location of cerebral infarction and the whole brain, and calculated the proportion of cerebral infarction (Xu et al. 2002; Zhang et al. 2017). Proportion of cerebral infarction = infarct site quality/whole brain quality × 100%.

### Terminal deoxynucleotidyl transferase-mediated dUTP nick-end labelling (TUNEL) assay

The standard paraffin block was prepared and a series of 6 μm thick sections were cut. After that, apoptosis was evaluated using TUNEL detection kit (Roche, Indianapolis, IN) following the manufacturer’s instructions, and was analysed under light microscope.

### Immunohistochemistry (IHC) staining

The expressions of HIF-1α, EPO and VEGFA were detected by IHC. The non-specific antigen was blocked by normal goat serum, and then brain tissue slices were incubated with primary antibody (rabbit-anti-rat, 1:100; Abcam Trading Co., Ltd., Shanghai, China) overnight at 4 °C. Then, the slices were incubated with secondary antibodies (goat-anti-rabbit, 1:500; Boster Co., Ltd., Wuhan, China). Finally, the slices were stained with DAB chromogenic reagent kit (ZLI-9018; Beijing Noble Ryder Technology Co., Ltd., Beijing, China) and haematoxylin staining solution, and were imaged under the microscope (magnification ×400; BX51T-PHD-J11; Olympus, Shinjuku, Japan). The proportion of positive cells was analysed by the true colour multi-functional cell image analysis management system (Image-Pro Plus; Media Cybernetics, Rockville, MD).

### Western blot analysis

Western blot was used to analyse the protein expressions of HIF-1α, EPO and VEGFA. Brain tissue proteins were collected, separated by electrophoresis, and then transferred to polyvinylidene difluoride membrane. The membrane was incubated overnight at 4 °C with anti-HIF-1α (ab1; Abcam Trading Co., Ltd., Shanghai, China), anti-EPO (ab226956; Abcam Trading Co., Ltd., Shanghai, China) and anti-VEGFA (ab1316; Abcam Trading Co., Ltd., Shanghai, China). The membrane was then incubated with secondary antibody: Goat anti-Mouse (BA1050; Boster Co., Ltd., Wuhan, China) or Goat anti-Rabbit (BA1054; Boster Co., Ltd., Wuhan, China). The internal reference β-actin (BM0627; Boster Co., Ltd., Wuhan, China) was used to calculate the relative expression of target protein.

### Components detection in brain tissue

After fasting for 12 h, six male Sprague-Dawley rats were given JCT-H (3.12 g/kg) by gavage for a single time, and the other six rats were given distilled water as blank control. After 0.5 h, the rats were killed and the brain tissue was taken. Brain tissue samples were homogenized with three times volume of normal saline. Supernatant (200 μL) was transferred to a 1.5 mL Eppendorf tube, 10 μL of internal standard solution (puerarin, 2 μg/mL) and 400 μL of methanol were added, and then centrifuged (10,000 rpm) for 10 min. The components in brain tissue were detected by HPLC–MS.

Chromatographic conditions: the column was InertSustain^®^C18 chromatographic column (3.0 mm × 100 mm, 3.0 µm); the column temperature was 35 °C; the mobile phase was acetonitrile (A)–0.1% formic acid aqueous solution (B), and elution method was gradient elution (0.00–3.50 min, 15% A; 3.51–6.00 min, 65% A; 6.01–11.00 min, 85% A); flow rate was 0.4 mL/min, sample injection volume was 10 µL.

Mass spectrometry conditions: the instrument was LCMS-8040 liquid mass spectrometer of Shimadzu (Kyoto, Japan); ion source was ESI, positive and negative ion mode; quantitative analysis adopted multiple reaction ion monitoring (MRM); heating module temperature was 400 °C; DL temperature was 250 °C; atomization gas flow rate was 3.0 L/min; dry gas flow rate was 15 L/min; ion source voltage was 4.0 kV.

### Molecular docking

The 3D structures of HIF-1α, EPO and VEGFA were obtained from RCSB-PDB database (https://www.rcsb.org/; PDB ID: 1LQB, 1EER, 4DEQ), and the component structures were download from PubChem database. In this study, PyRx-Virtual Screening Tool was applied for docking. During the docking simulation, the protein was kept rigid and the ligand moved. The parameters were kept at default values. For each ligand, the lowest binding energy and different conformations were recorded. Discovery Studio software was used for the final visualization.

### Statistical analysis

Data were presented as the mean ± standard deviation (*x̅*±*s*). The *t*-test was used to compare the mean value of two independent samples, and *p* < 0.05 was considered statistically significant.

## Results

### Network establishment

**Figure 1.**
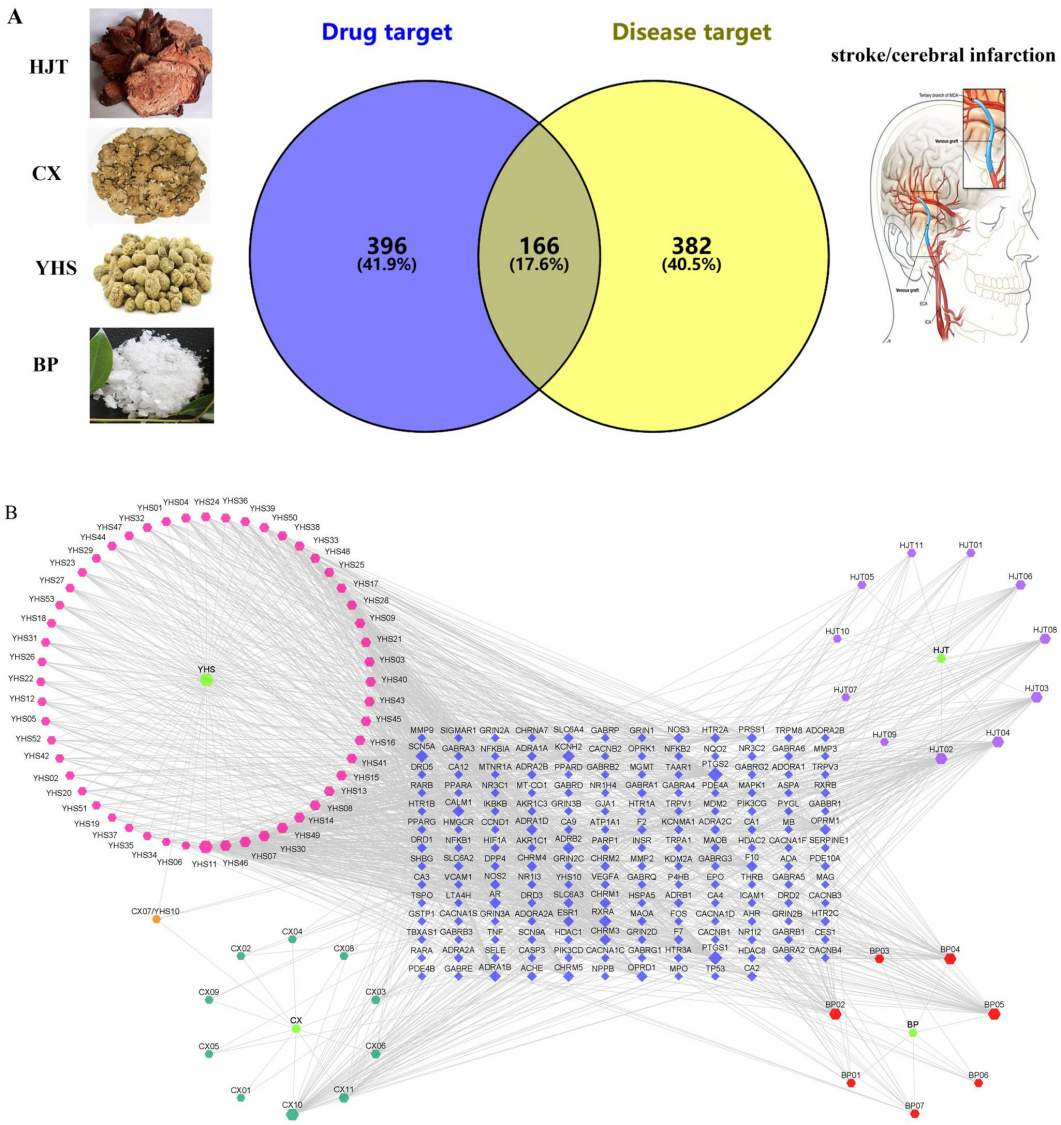
Network establishment. (A) Venn diagram of drug targets and disease targets. (B) ‘Drug-component-common target’ network. Blue indicates targets, green indicates drugs and other colours indicate active compounds.

In our study, according to the search conditions, 81 components were retrieved from JCT (Table 1). After searching for the targets of the four herbs of JCT, we found that the targets of each herb obviously overlapped. Therefore, 562 potential targets of 81 components from the four herbs were extracted after removing the duplication. To identify disease targets, 548 targets related to ICS were retrieved from TCMSP, TTD and DrugBank databases. In the VENNY 2.1 website, component-related targets and ICS-related targets were submitted as two independent sets. Then, a Venn diagram was constructed to analyse the relationship between the two sets and calculate the overlapping data. Finally, a total of 166 common targets were filtered out, as shown in Figure 1(A). A ‘drug-component-common target’ network was constructed, and the size and colour depth of the nodes were set according to the degree value (Figure 1(B)). The more important the node was, the larger its size and the darker its colour would be in the network. There were 59 components with a degree greater than or equal to 10 (Table 1).

**Table 1.** The active components among the JCT for network analysis.

Herb	Node name	Molecule name	Degree	Source
Borneolum syntheticum	BP01	Camphor	9	TCMIP
	BP02	dl-Isoborneol	45	TCMIP
	BP03	Asiatic acid	13	TCMSP, TCMIP
	BP04	Borneol	45	TCMIP
	BP05	Dipterocarpol	43	TCMSP, TCMIP
	BP06	Erythrodiol	6	Chen and Feng (2013)
	BP07	Bornyl acetate	12	TCMSP, TCMIP
*Ligusticum chuanxiong* Hort. (Umbelliferae)	CX01	FA	5	TCMSP
	CX02	Perlolyrine	4	TCMSP
	CX03	Senkyunone	10	TCMSP
	CX04	Wallichilide	4	TCMSP
	CX05	Mandenol	3	TCMSP
	CX06	Myricanone	14	TCMSP
	CX07/YHS10	Sitosterol	6	TCMSP
	CX08	Tetramethylpyrazine	5	TCMSP, TCMIP
	CX09	Ligustilide	3	TCMSP, TCMIP
	CX10	Ferulic acid	48	TCMSP
	CX11	Chlorogenic acid	22	TCMSP
*Rhodiola crenulata* (Hook. f. et Thoms.) H. Ohba (Crassulaceae)	HJT01	Caffeic acid	12	TCMIP
	HJT02	Gallic acid	12	TCMIP
	HJT03	Ethyl gallate	7	Nan et al. (2018)
	HJT04	Salidroside	18	Sun et al. (2012)
	HJT05	Tyrosol	9	Sun et al. (2012)
	HJT06	Hydroxytyrosol	23	Chung et al. (2017)
	HJT07	Hydroquinone	3	Wang et al. (2007)
	HJT08	Kaempferol	59	Ma et al. (2017)
	HJT09	Rosavin	8	Mudge et al. (2012)
	HJT10	Rosarin	6	Mudge et al. (2012)
	HJT11	Rosin	8	Mudge et al. (2012)
*Corydalis yanhusuo* (Y. H. Chou et Chun C. Hsu) W. T. Wang ex Z. Y. Su et C. Y. Wu (Papaveraceae)	YHS01	Berberine	17	TCMSP
	YHS02	Coptisine	10	TCMSP
	YHS03	Cryptopin	23	TCMSP
	YHS04	Dihydrochelerythrine	15	TCMSP
	YHS05	Dihydrosanguinarine	11	TCMSP
	YHS06	Sanguinarine	4	TCMSP
	YHS07	Scoulerin	32	TCMSP
	YHS08	Cavidine	27	TCMSP
	YHS09	(*R*)-Canadine	32	TCMSP
	CX07/YHS10	Sitosterol	6	TCMSP
	YHS11	Tetrahydropalmatine	31	TCMSP
	YHS12	(–)-alpha-*N*-Methylcanadine	11	TCMSP
	YHS13	Capaurine	26	TCMSP
	YHS14	Clarkeanidine	28	TCMSP
	YHS15	Corydaline	24	TCMSP
	YHS16	Corydalmine	23	TCMSP
	YHS17	Corydine	20	TCMSP
	YHS18	Corynoline	12	TCMSP
	YHS19	Corynoloxine	8	TCMSP
	YHS20	Methyl-[2-(3,4,6,7-tetramethoxy-1-phenanthryl)ethyl]amine	9	TCMSP
	YHS21	Dehydrocavidine	22	TCMSP
	YHS22	Dehydrocorybulbine	11	TCMSP
	YHS23	Dehydrocorydaline	13	TCMSP
	YHS24	Dehydrocorydalmine	15	TCMSP
	YHS25	Demethylcorydalmatine	19	TCMSP
	YHS26	13-Methyldehydrocorydalmine	11	TCMSP
	YHS27	Fumaricine	12	TCMSP
	YHS28	Izoteolin	20	TCMSP
	YHS29	Isocorybulbine	18	TCMSP
	YHS30	Leonticine	32	TCMSP
	YHS31	13-Methylpalmatrubine	12	TCMSP
	YHS32	N-methyllaurotetanine	15	TCMSP
	YHS33	Norglaucing	19	TCMSP
	YHS34	Pontevedrine	5	TCMSP
	YHS35	Pseudocoptisine	7	TCMSP
	YHS36	Pseudoprotopine	16	TCMSP
	YHS37	Saulatine	8	TCMSP
	YHS38	Stylopine	18	TCMSP
	YHS39	Tetrahydrocorysamine	16	TCMSP
	YHS40	Tetrahydroprotopapaverine	23	TCMSP
	YHS41	ST057701	24	TCMSP
	YHS42	2,3,9,10-Tetramethoxy-13-methyl-5,6-dihydroisoquinolino[2,1-b]isoquinolin-8-one	10	TCMSP
	YHS43	Stigmasterol	23	TCMSP
	YHS44	Palmatine	15	TCMSP
	YHS45	Fumarine	23	TCMSP
	YHS46	Isocorypalmine	33	TCMSP
	YHS47	Bicuculline	16	TCMSP
	YHS48	Bulbocapnine	19	TCMSP
	YHS49	Quercetin	53	TCMSP
	YHS50	Columbamine	17	TCMSP
	YHS51	Oxoglaucine	9	TCMSP
	YHS52	Epiberberine	11	TCMSP
	YHS53	Jatrorrhizine	12	TCMSP

### Network analysis

**Figure 2.**
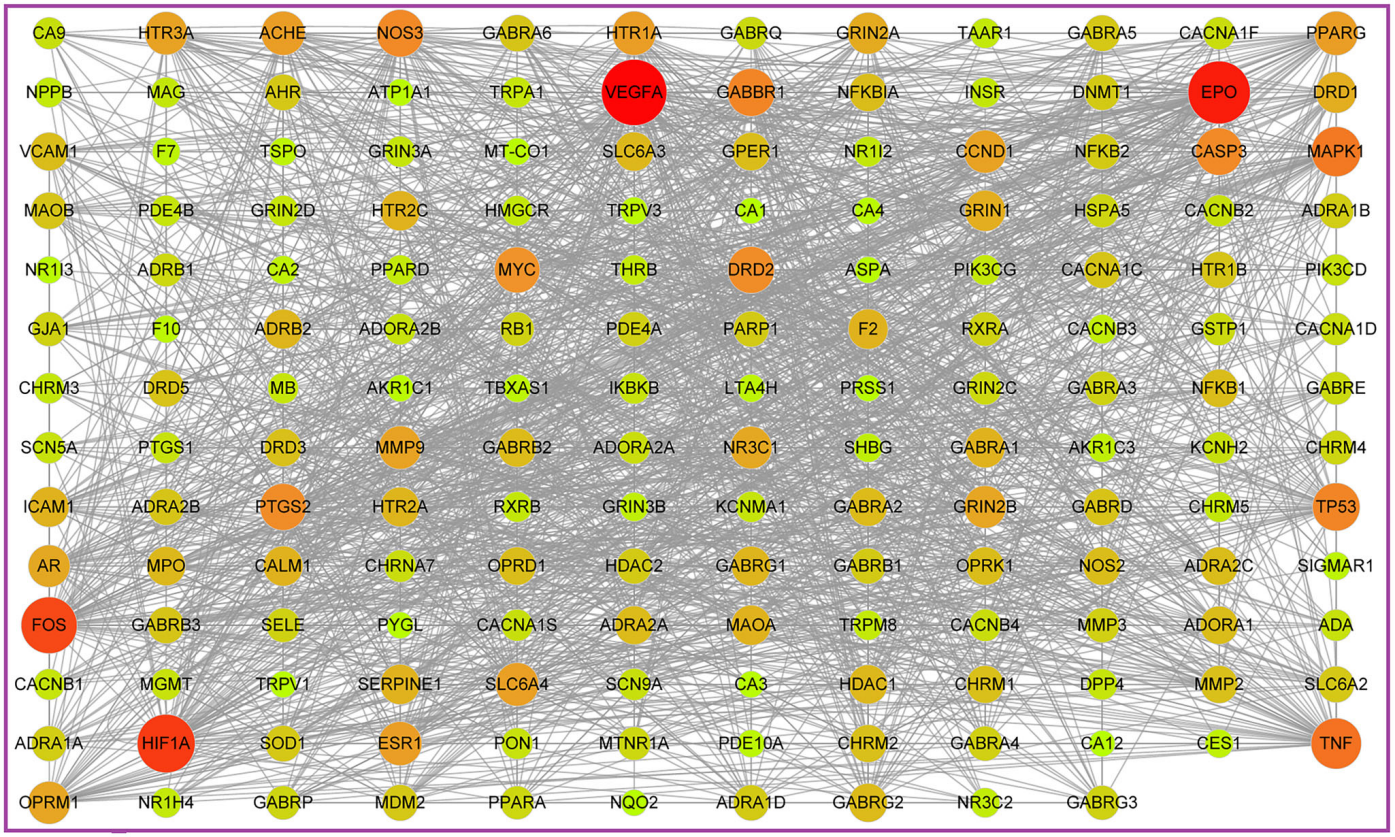
PPIs network analysis.

The PPIs was obtained by integrating the STRING 11.0 and GenCLiP 3 databases. The ‘PPIs network’ was visualized in Cytoscape 3.7.2, as shown in Figure 2. The larger the shape of a node and the closer its colour was to red, the more important the target represented by the node would be. We found VEGFA, EPO and HIF-1α (HIF1A) were more important than others and closely related to other targets in the network. With degree as the index, the core targets were obtained by one-half median step-by-step screening and modelled by the MCODE analysis. Therefore, 10 core targets were obtained as EPO, HIF-1α, VEGFA, MYC, FOS, TP53, CASP3, TNF, PTGS2, and MAPK1. Among these targets, EPO, HIF-1α and VEGFA got the highest degree value. Overall, it was speculated that EPO, HIF-1α and VEGFA played the key role in the treatment of ICS by JCT.

**Figure 3.**
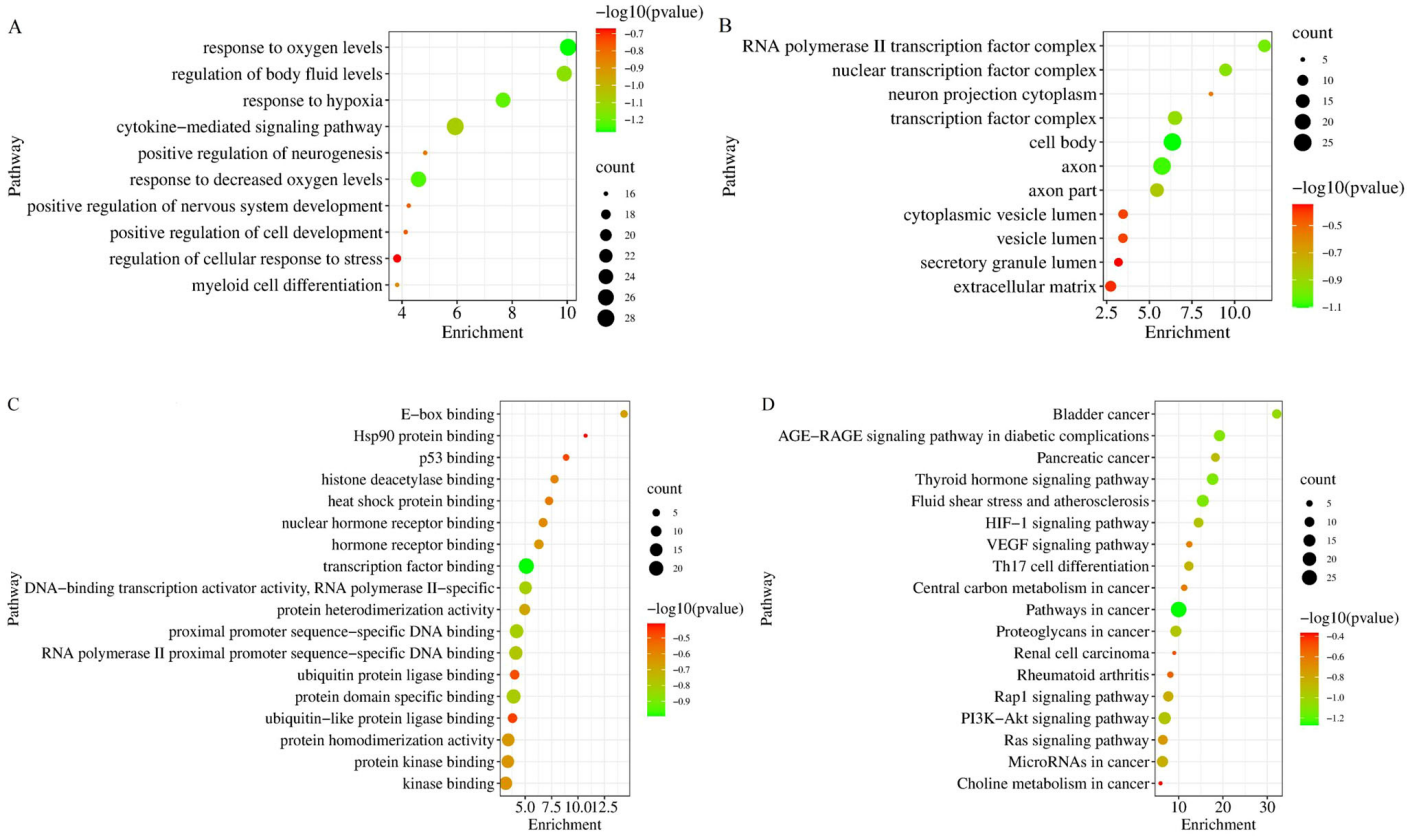
Results of GO enrichment analysis. (A) BiP analysis. (B) CC analysis. (C) MF analysis. (D) KEGG pathway analysis.

Additionally, GO enrichment analysis and KEGG pathway analysis of the 166 common targets were performed in the Metascape database. Through GO enrichment analysis, we could know which gene functions the differential targets were mainly involved in. GO analysis results showed HIF-1α, EPO and VEGFA were involved in 552 BiPs, and the first 10 BiPs are listed in Figure 3(A). These BiPs included response to oxygen levels, response to hypoxia, and positive regulation of nervous system, etc. These results indicated that HIF-1α, EPO and VEGFA had the function of hypoxia regulation and neuromodulation. In addition, improving hypoxia ischaemia and neural function was an important way to treat ICS, and HIF-1α, EPO and VEGFA played an important role in the pathogenesis and treatment of cerebral ischaemia (Novikov and Levchenkova 2020; Dong et al. 2021; Ma et al. 2022). Therefore, it was speculated that the anti-ICS effects of JCT could be achieved by regulating HIF-1α/EPO/VEGFA.

**Figure 4.**
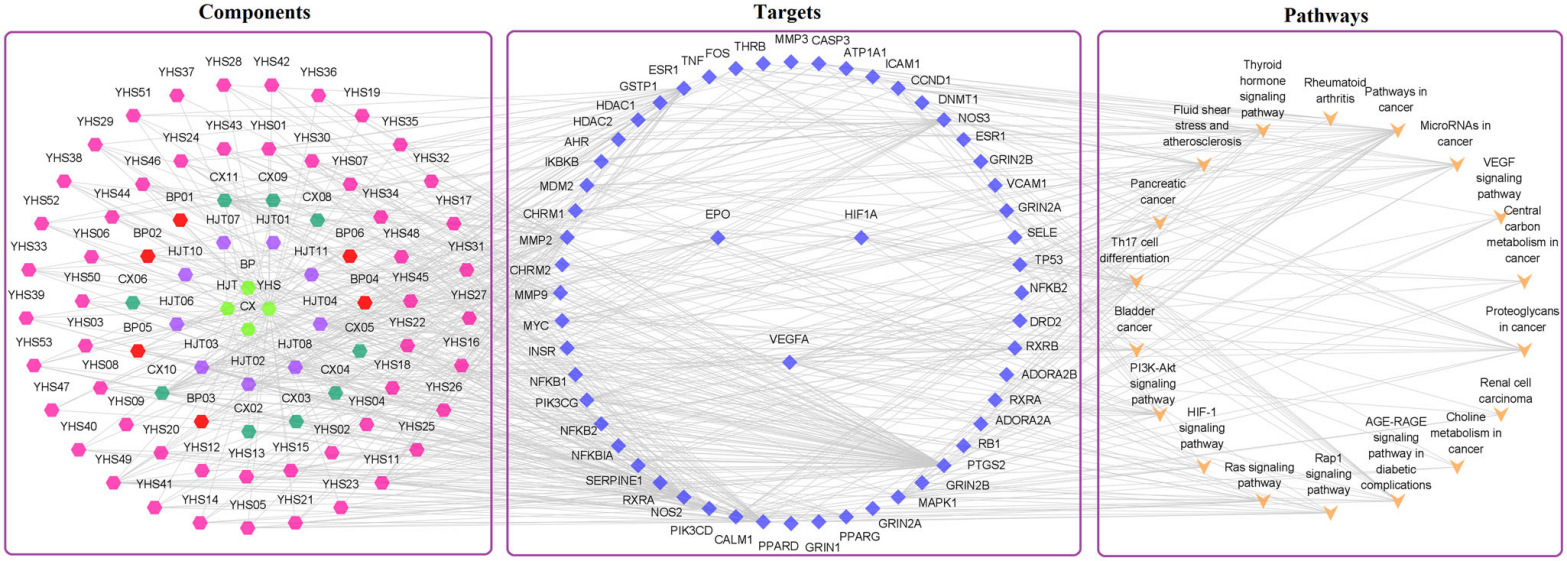
‘Component-target-pathway’ network.

There were 11 CCs related to HIF-1α, EPO and VEGFA, with a frequency greater than or equal to 3 (Figure 3(B)). Axon appeared most frequently, followed by cell body and transcription factor complex. Among the MFs that occurred at least three times, 18 MFs were involved in HIF-1α, EPO and VEGFA (Figure 3(C)). Then transcription factor binding appeared most frequently, followed by protein domain specific binding and proximal promoter sequence-specific DNA binding. The analysis results of KEGG pathway revealed that HIF-1α, EPO and VEGFA participated in 18 signalling pathways. They were markedly enriched in HIF-1 pathway, VEGF pathway and PI3K-Akt pathway, etc. (Figures 3(D) and 4).

There were 81 compounds that regulated these core targets were considered as the effective compounds, and the top 10 components with the highest degree value were ferulic acid (CX10), tetrahydropalmatine (YHS11), salidroside (HJT04), kaempferol (HJT08), quercetin (YHS49), gallic acid (HJT02), chlorogenic acid (CX11), ethyl gallate (HJT03), dipterocarpol (BP05) and corydalmine (YHS16) (Figure 4).

### Effects of JCT on neurobehavioural scores

Forty-eight hours after modelling, the model group had higher neurological behaviour scores, and compared with the sham group, the increased level had significant difference (*p* < 0.01). Compared with the model group, the neurobehavioural scores of the different doses of JCT were significantly lower (*p* < 0.05 or *p* < 0.01) in the injured rats. The scores of JCT-H and JCT-M groups were lower than those in positive drug group, as shown in Table 2. It was suggested that JCT had the effects of repairing nerve function injury in rats, and the effects were better than that of nimodipine to a certain extent.

**Table 2.** Neurobehavioural score (*n*= 10).

Group	Dose (g/kg)	Neurological score
Sham	–	0.00 ± 0.00
Model	–	3.50 ± 1.00##
JCT-H	3.120	2.00 ± 0.58**
JCT-M	1.560	2.10 ± 0.77**
JCT-L	0.780	2.60 ± 0.61*
Positive drug	0.012	2.15 ± 0.67**

##*p*< 0.01 compared with the sham group.

*p< 0.05, **p< 0.01 compared with the model group.

### The ratio of cerebral infarction

**Figure 5.**
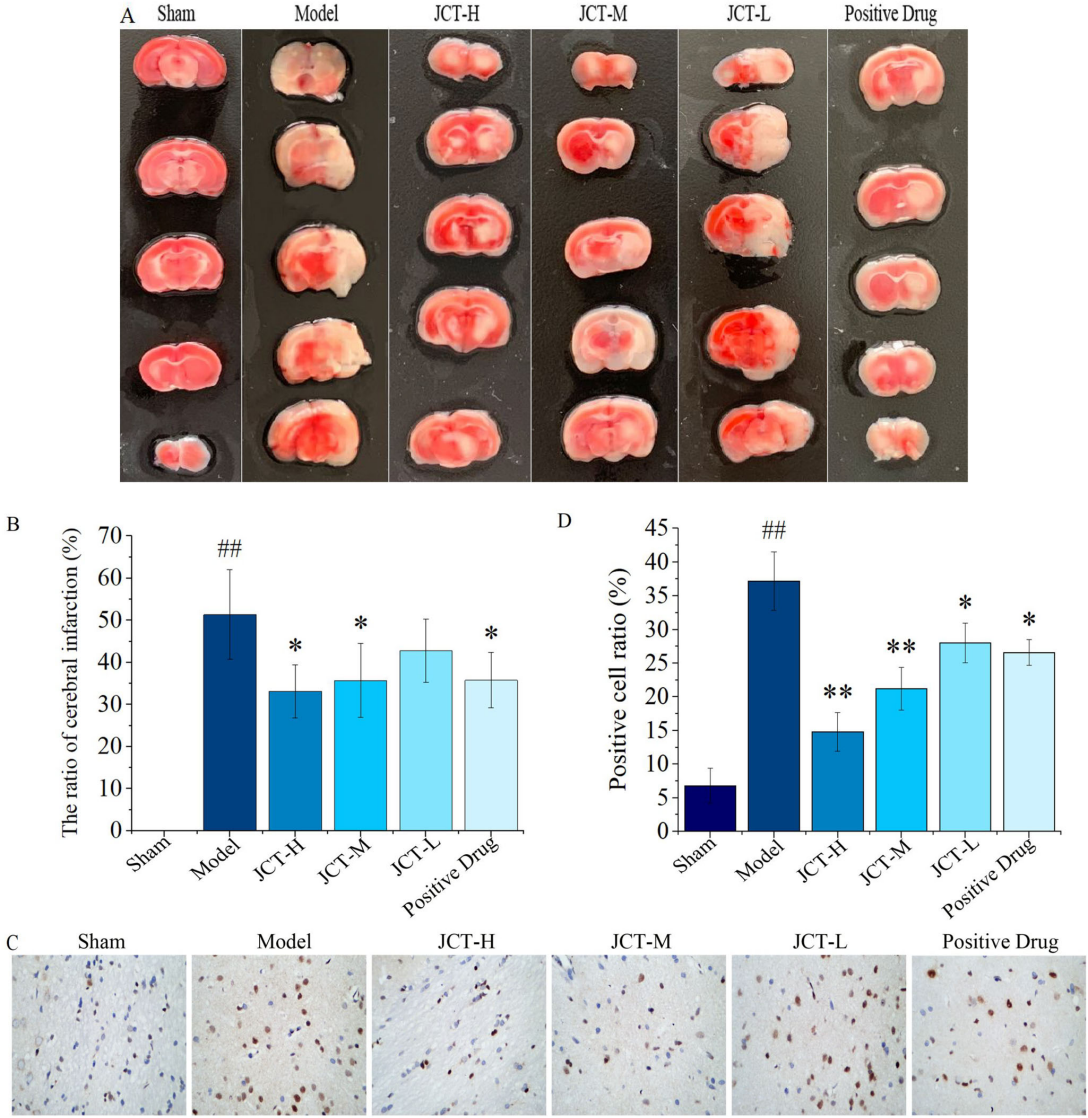
Therapeutic effect of JCT on cerebral ischaemic. (A) The representative images of TTC staining. (B) Quantitative analysis of TTC staining. (C) Images of TUNEL detection (magnification ×400). (D) Quantitative analysis of TUNEL staining (*n*= 5). ^##^*p*< 0.01 compared with the sham group; *p< 0.05 and **p< 0.01 compared with the model group.

Cerebral infarction was assessed by TTC staining, and the infarct area of brain tissue in rats showed a white ischaemic state. No cerebral infarction was found in the sham group. Compared with the sham group, the infarct region of the model group was significantly increased (51.35 ± 10.64%, *p* < 0.01). After JCT intervention, the area of cerebral infarction in each treatment group of JCT was reduced from 42.73 ± 7.51 to 33.13 ± 6.28%. The degree of reduction in the JCT-H and JCT-M groups was significantly different from that in the model group (*p* < 0.05), and was better than that in the positive drug group (35.78 ± 6.56%) (Figure 5(A,B)). The above results revealed that JCT could effectively reduce infarct volume and improve cerebral ischaemia state in rats.

### Effects of JCT on neuronal apoptosis in cerebral ischaemic area

TUNEL staining was used to detect the protective effects of JCT on cell apoptosis. After TUNEL staining, the apoptotic neurons in the cortical area of rats contracted with brown colour (Figure 5(C)). The results of quantitative analysis are shown in Figure 5(D). It showed that ischaemia injury resulted in a significantly increased rate of TUNEL positive cells in the pMCAO model group (37.20 ± 4.32%, *p* < 0.01), while there were few TUNEL positive cells detected in the sham group. Meanwhile, the cell apoptosis rate decreased significantly in the JCT (28.0 ± 2.92 to 14.80 ± 2.86%, *p* < 0.05 or *p* < 0.01) and positive drug (26.60 ± 1.95%, *p* < 0.05) groups, compared with those in the model group. In addition, the cell apoptosis rate in the JCT-H and JCT-M groups was significantly less than that of the positive drug group. The results indicated that JCT could protect nerve cells from injury.

### Effects of JCT on expressions of HIF-1α/EPO/VEGFA in IHC

**Figure 6.**
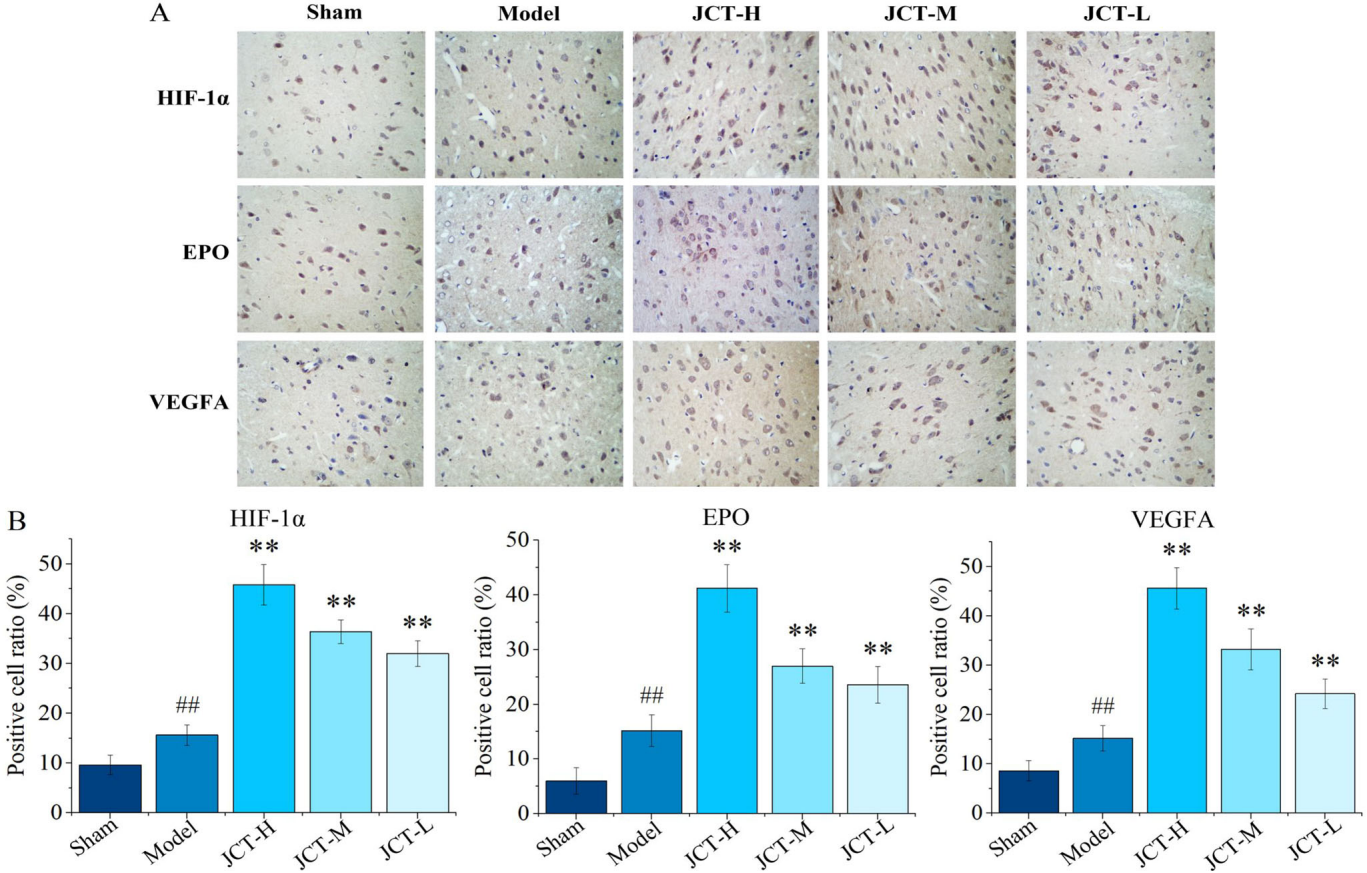
Effects of JCT on expression of HIF-1α, EPO and VEGFA in IHC. (A) Images of IHC (magnification ×400). (B) Immunohistochemical analysis of HIF-1α, EPO and VEGFA in cerebral ischaemia region (*n*= 5). ^##^*p*< 0.01 compared with the sham group; **p< 0.01 compared with the model group.

IHC was performed to determine whether JCT could affect the expression of HIF-1α/EPO/VEGFA. The results of IHC showed that the expression of HIF-1α, EPO and VEGFA in the area of cerebral ischaemia injury increased significantly after modelling (*p* < 0.01) (Figure 6). Furthermore, the expression of HIF-1α, EPO and VEGFA in the JCT groups was markedly increased in comparison with the model group (*p* < 0.01). Therefore, JCT played a role in the treatment of ICS by activating the HIF-1α/EPO/VEGFA pathway.

### Effects of JCT on protein levels of HIF-1α/EPO/VEGFA in Western blots

**Figure 7.**
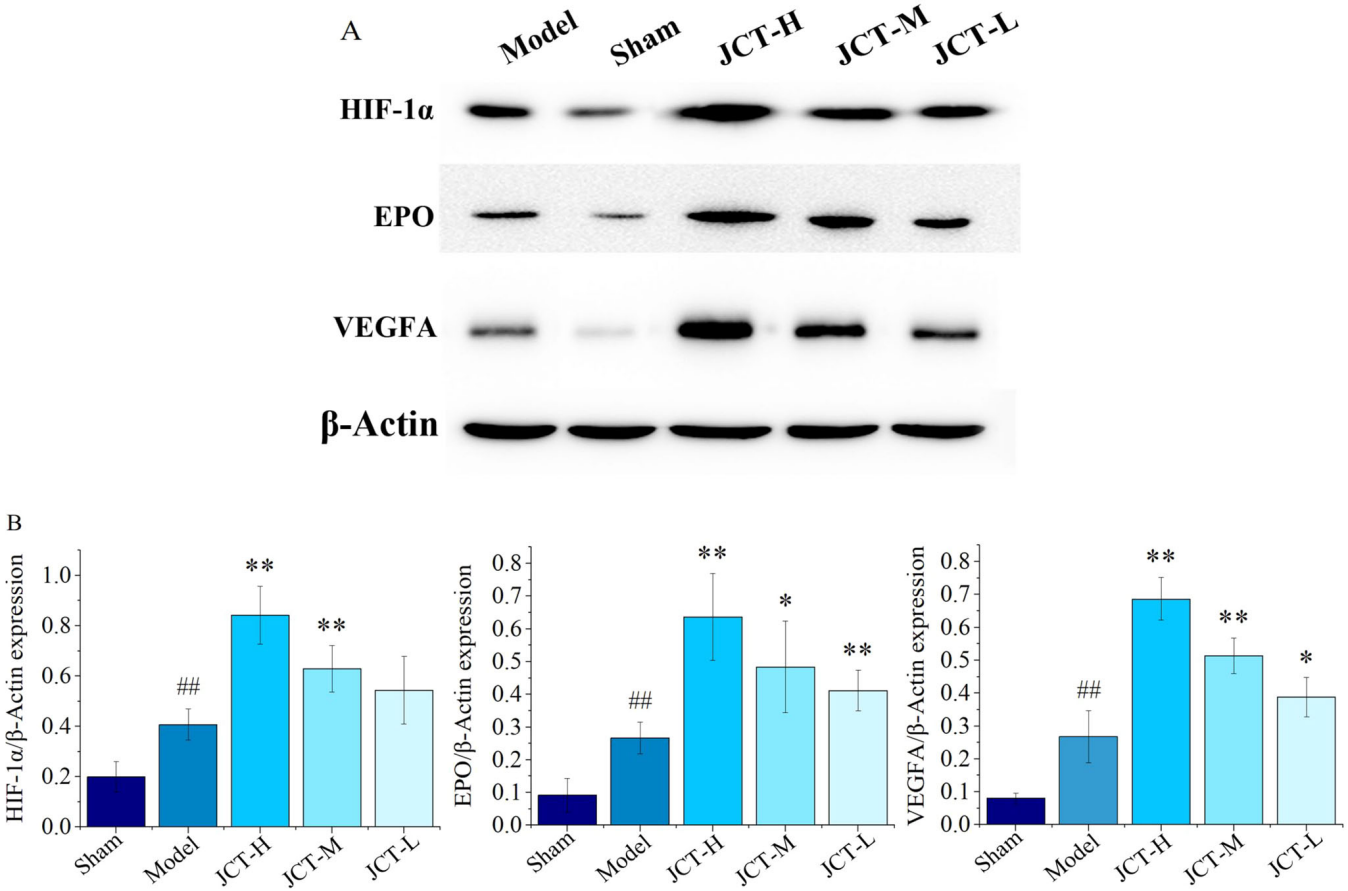
Effects of JCT on the levels of HIF-1α, EPO and VEGFA. (A) Cropped Protein bands. (B) Quantitative analysis of HIF-1α/β-actin, EPO/β-actin and VEGFA/β-actin (*n*= 5). ^##^*p*< 0.01 compared with the sham group; *p< 0.05 and **p< 0.01 compared with the model group.

The results of Western blots are shown in Figure 7. The protein levels of HIF-1α, EPO and VEGFA were less expressed in the sham group (0.20 ± 0.06; 0.09 ± 0.05; 0.08 ± 0.02). After the establishment of pMCAO model, the protein expression levels of HIF-1α, EPO and VEGFA were significantly increased (0.41 ± 0.06; 0.27 ± 0.05; 0.27 ± 0.08) (*p* < 0.01). After treatment with different doses of JCT, the expression of EPO (0.41 ± 0.06 to 0.64 ± 0.13) and VEGFA (0.39 ± 0.06 to 0.69 ± 0.07) was significantly higher than those in the model group (*p* < 0.01 or *p* < 0.05). Compared with the model group, the protein levels of HIF-1α in each treatment group of JCT were increased, but only the increase in the JCT-H (0.84 ± 0.11) and JCT-M (0.63 ± 0.09) groups was statistically significant (*p* < 0.01). In addition, the expression of these proteins depended on the dose of JCT. The higher the dose of JCT, the higher their expression. It could be seen that HIF-1α, EPO and VEGFA played an important role in the treatment of ICS rats by JCT. Collectively, these data highlighted the role of the HIF-1α/EPO/VEGFA signalling pathway in the regulation of ICS by JCT.

### Components detection in brain tissue

The components in brain tissue of rats after intragastric administration of JCT-H were detected. The results showed that the main components detected were gallic acid (HJT02), salidroside (HJT04), chlorogenic acid (CX11), ethyl gallate (HJT03), ferulic acid (CX10) and tetrahydropalmatine (YHS11) (Table 3). Taking puerarin as the reference, the contents of HJT02, HJT04, CX11, HJT03, CX10 and YHS11 were measured as 111.07, 108.73, 44.52, 14.70, 8.46 and 10.49 ng/g (Table 4). These detected components were all ranked in the top 10 among the active components screened by the network pharmacology, indicating the accuracy of network prediction results.

**Table 3.** MS parameters of components.

Components	*t*_R_ (min)	Ion mode	MRM	Q1 pre bias (V)	Collision energy (V)	Q3 pre bias (V)
Gallic acid	1.736	ESI(–)	169.10 → 125.05	18	17	20
Salidroside	2.183	ESI(–)	345.20 → 299.15	23	11	29
Chlorogenic acid	2.89	ESI(–)	353.20 → 191.05	24	18	30
Puerarin	3.367	ESI(–)	415.20 → 267.00	28	33	25
Ethyl gallate	5.962	ESI(–)	197.10 → 124.00	21	23	21
Ferulic acid	6.498	ESI(–)	193.10 → 134.00	21	14	25
Tetrahydropalmatine	7.037	ESI(+)	355.60 → 192.10	–25	–24	–14

**Table 4.** The contents of compounds were measured in the brain tissue of six male Sprague-Dawley rats.

Sample number	Content (ng/g)					
	Gallic acid	Salidroside	Chlorogenic acid	Ethyl gallate	Ferulic acid	Tetrahydropalmatine
1	100.17	45.97	45.52	14.52	8.08	7.59
2	119.85	135.88	44.07	14.84	8.58	11.47
3	93.31	42.64	41.79	14.32	7.99	7.64
4	117.47	146.14	45.08	14.83	8.69	12.57
5	120.53	135.18	45.13	14.90	8.76	11.46
6	115.12	146.59	45.53	14.77	8.67	12.20
Mean	111.07	108.73	44.52	14.70	8.46	10.49

### Docking analysis of the correlation between active components and HIF-1α/EPO/VEGFA

After confirming the role of JCT in activating the HIF-1α/EPO/VEGFA pathway, we further explored the interactive activities between JCT compounds with HIF-1α/EPO/VEGFA by docking analysis. Molecular docking was performed on six main bioactive components, which were derived from the top 10 compounds based on the degree value in network pharmacology and detected in the brain tissue. The results showed that the six active components had good spatial and energy matching with HIF-1α, EPO and VEGFA at the same time (Figure 8). In addition, the results revealed the hydrogen bond and hydrophobic interaction between molecule and the receptor protein (Figure 8). The above results suggested that the active components of JCT could realize relevant BiPs by regulating key targets such as HIF-1α, EPO and VEGFA. This view further supported our previous experimental conclusions, indicating that the research method was scientific and accurate to a certain extent. Overall, these findings not only laid the foundation for the later pharmacological studies on these natural compounds, but also provided more evidence for our previous conclusions.

Figure 8.Docking analysis. (A) Interactions between EPO and ligands. (B) Interactions between HIF-1α and ligands. (C) Interactions between VEGFA and ligands.

**Figure:**
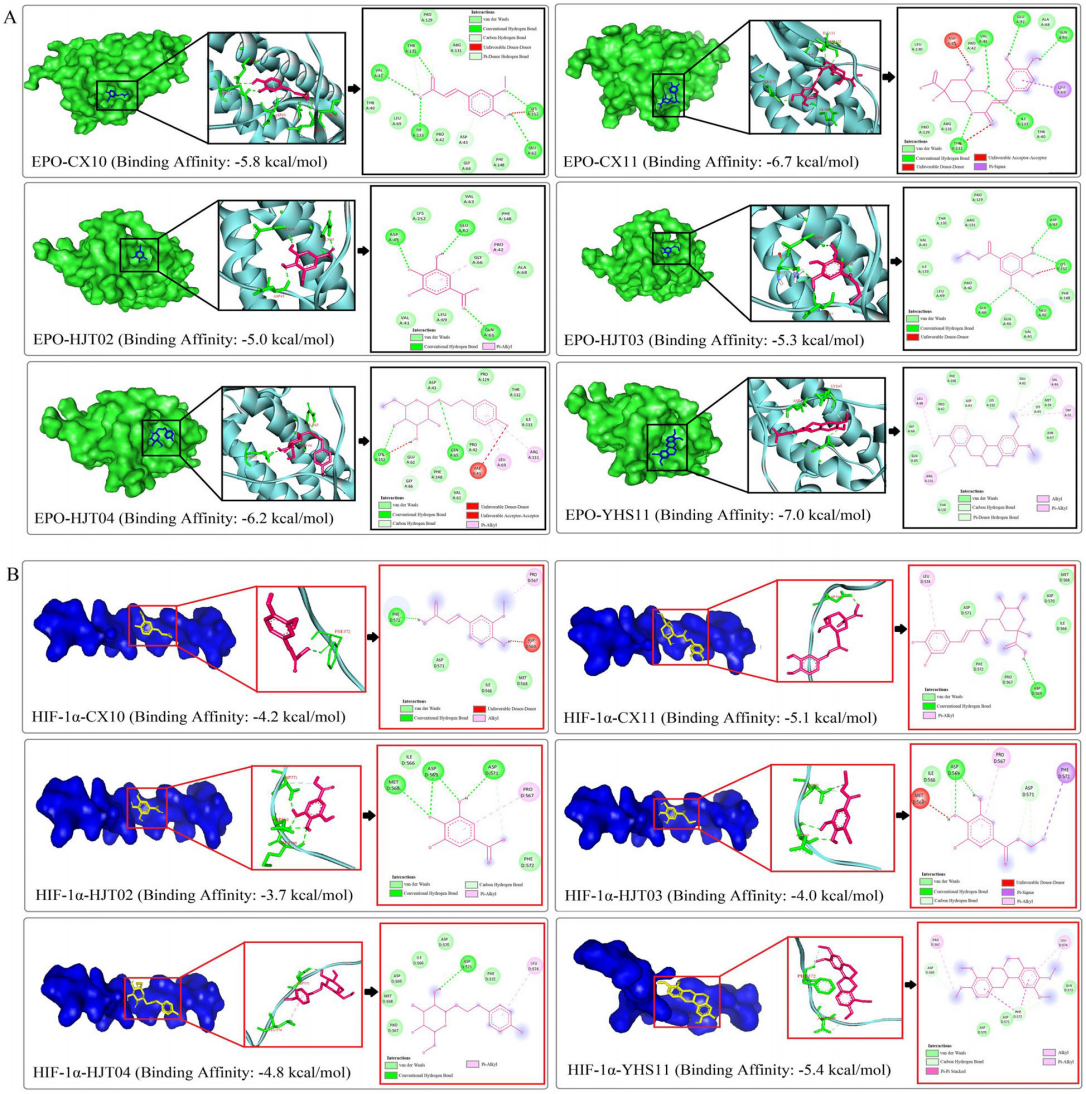

**Figure:**
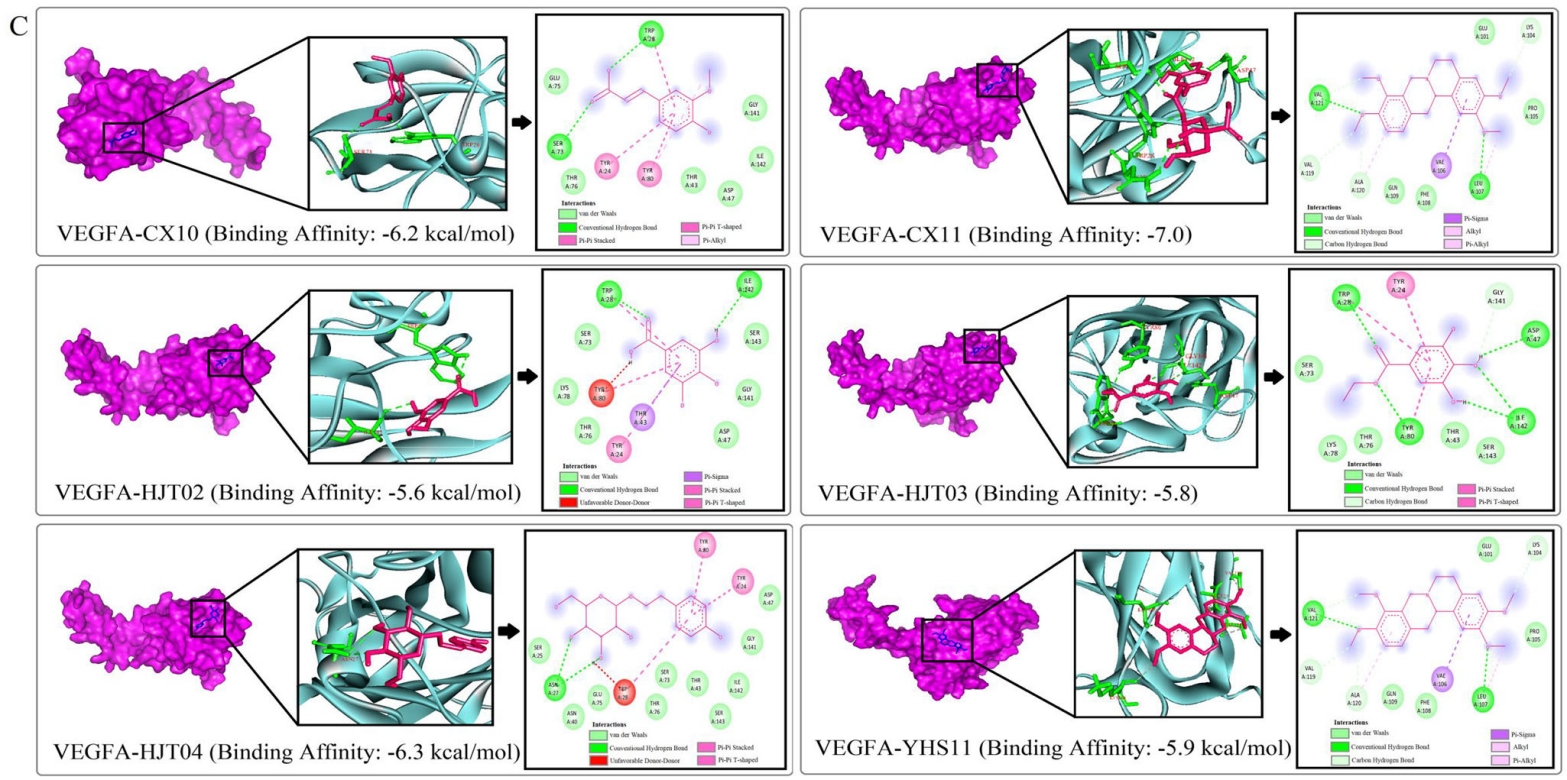

## Discussion

ICS is mainly caused by ischaemia and hypoxia due to the disorder of cerebral blood supply. Its main manifestations are neurocognitive function and limb disorders. The current therapies for ICS are tissue plasminogen activator and mechanical thrombectomy, which re-establishes blood circulation to the brain but offers no neuroprotective effects. Given that, new therapeutic strategies are increasingly needed due to the ineffective control of ICS.

Although the action mechanisms of TCM are not quite clarified owing to its characteristics of multi-components, multi-targets and multi-pathways, accumulating evidence have confirmed that TCM is clinically useful for anti-ICS. Revealing the intrinsic mechanisms can provide the scientific explanations for the clinical application of TCM. With the development of network pharmacology, modern pharmacological investigations have more tools to explore the complex mechanisms of TCM (Zhou et al. 2020; Chen et al. 2022).

As a Chinese medicine prescription, there was a lack of explanation for the anti-ICS of JCT. Therefore, we planned to conduct a series of studies to explore the underlying mechanisms of JCT’s anti-ICS effects and find useful natural components based on the major ICS-targeted organ. In this present study, we investigated the potential effects and intrinsic mechanisms of JCT on ICS by a combination of bioinformatics and modern pharmacology, and revealed that the therapeutic role of JCT on ICS was mediated by activating the HIF-1α/EPO/VEGFA pathway. HIF-1α, EPO and VEGFA had the gene functions such as response to oxygen levels, response to hypoxia and positive regulation of nervous system. JCT could up-regulate the expression of HIF-1α/EPO/VEGFA pathway and promote it to play a therapeutic role by participating in the BiPs of ischaemia and hypoxia. Vascular endothelial growth factor (VEGF) is a potent inducer of erythropoietin (EPO) (Greenwald et al. 2019). EPO plays an important role in angiogenesis induced by ischaemia through the upregulation of VEGF and its receptor system (Liu et al. 2020). EPO and vascular VEGF are the target genes for hypoxia inducible factor-1α (HIF-1α), and HIF-1α is a master regulator of VEGF and EPO (Jin et al. 2020; Li et al. 2020; Zhu et al. 2020). Activated HIF-1α can regulate anaerobic metabolism by inducing the expression of its critical downstream factors EPO and VEGF to help cells adapt to hypoxia and protect neurons from ischaemic insults during ICS (Zhang et al. 2014; Jin et al. 2020; Li et al. 2020).

The pMCAO model was an appropriate animal model to simulate clinical ICS. Although it could not fully reflect the complex state of ICS in human body, it was required for understanding the exact pathophysiological mechanisms of ICS. In order to verify JCT’s ICS-inhibiting effects, the pMCAO model experiments *in vivo* were performed. These three doses of JCT were mainly determined by human-mouse dose conversion formula and our previous study (Tao et al. 2017; Zhang et al. 2019). JCT-M in this work was an equivalent regular dose used in clinic, and the JCT-H and JCT-L were set up to better explore the therapeutic effects of JCT. In this study, JCT displayed obvious therapeutic effects on ICS, which could not only re-establish blood circulation to reduce the symptoms of cerebral infarction in ICS rats, but also offer neuroprotective effects to improve the nerve function of ICS rats. Overall, a combined ameliorative effect on ICS of JCT was observed in our study.

In our study, we retrieved 81 active compounds of JCT as the potential effective components by network pharmacology analysis. Actually, the main bioactive components detected in rat brain tissue were gallic acid, salidroside, chlorogenic acid, ethyl gallate, ferulic acid and tetrahydropalmatine. Interestingly, these six components were all ranked in the top 10 among the above-mentioned 81 compounds. Therefore, our results supported the view of network analysis. For further explaining the interplay between pharmacodynamic substances and core therapeutic targets, computational molecular docking was performed and showed how compounds interacted with HIF-1α/EPO/VEGFA. In fact, a conclusion that JCT’s ICS-inhibiting effects were mediated by activating the HIF-1α/EPO/VEGFA signalling pathway could already be made *in vivo* experiment. Even though, the most crucial purpose of our still conducting docking analysis was to strengthen the reliability of the conclusion and provide a relatively objective idea for further research on the material basis of JCT anti-ICS.

## Conclusions

Our study explored the potential effects and intrinsic mechanisms of JCT against ICS by a comprehensive investigation that integrated network pharmacology, mass spectrometry, molecular docking, *in vivo* functional experiments and molecular biology. The results indicated that JCT could treat ICS by reducing the symptoms of cerebral infarction and enhancing neuroprotective effects, and its action mechanisms were associated with the activation of the HIF-1α/EPO/VEGFA pathway. The research in this article provided a scientific explanation for the clinical application of JCT and the basis for further research into the bioactive components and compounds of JCT against ICS. Further, the research model presented in this paper provided some ideas for the research of modern Chinese medicine prescription.
